# Analysis of the Anti-Cancer Effects of Cincau Extract (*Premna oblongifolia* Merr) and Other Types of Non-Digestible Fibre Using Faecal Fermentation Supernatants and Caco-2 Cells as a Model of the Human Colon

**DOI:** 10.3390/nu9040355

**Published:** 2017-04-03

**Authors:** Samsu U. Nurdin, Richard K. Le Leu, Graeme P. Young, James C. R. Stangoulis, Claus T. Christophersen, Catherine A. Abbott

**Affiliations:** 1 School of Biological Sciences, Flinders University, Adelaide, SA 5042, Australia; samsu.udayana@fp.unila.ac.id (S.U.N.); james.stangoulis@flinders.edu.au (J.C.R.S.); 2 Department of Agricultural Product Technology, Lampung University, Bandar Lampung 35145, Indonesia; 3 Flinders Centre for Innovation in Cancer, Adelaide, SA 5042, Australia; Richard.LeLeu@sa.gov.au (R.K.L.L.); graeme.young@flinders.edu.au (G.P.Y.); 4 CSIRO Food and Nutrition, Adelaide, SA 5000, Australia; c.christophersen@ecu.edu.au; 5 School of Medical and Health Sciences, Edith Cowan University, Joondalup, WA 6027, Australia

**Keywords:** dietary fibre, colorectal cancer, fermentation, cincau, short chain fatty acids

## Abstract

Green cincau (*Premna oblongifolia* Merr) is an Indonesian food plant with a high dietary fibre content. Research has shown that dietary fibre mixtures may be more beneficial for colorectal cancer prevention than a single dietary fibre type. The aim of this study was to investigate the effects of green cincau extract on short chain fatty acid (SCFA) production in anaerobic batch cultures inoculated with human faecal slurries and to compare these to results obtained using different dietary fibre types (pectin, inulin, and cellulose), singly and in combination. Furthermore, fermentation supernatants (FSs) were evaluated in Caco-2 cells for their effect on cell viability, differentiation, and apoptosis. Cincau increased total SCFA concentration by increasing acetate and propionate, but not butyrate concentration. FSs from all dietary fibre sources, including cincau, reduced Caco-2 cell viability. However, the effects of all FSs on cell viability, cell differentiation, and apoptosis were not simply explainable by their butyrate content. In conclusion, products of fermentation of cincau extracts induced cell death, but further work is required to understand the mechanism of action. This study demonstrates for the first time that this Indonesian traditional source of dietary fibre may be protective against colorectal cancer.

## 1. Introduction

Colorectal cancer (CRC) incidence is rising significantly in most countries due to increasing prosperity [1]. Compounds such as short chain fatty acids (SCFAs), which are produced by bacterial fermentation of undigested dietary fibre, are capable of inhibiting cancer in vitro and in vivo [2,3,4,5,6]. The type of dietary fibre consumed influences the proportion and distribution of SCFAs in the gastrointestinal tract [7,8,9,10]. Rapidly fermentable dietary fibre is fermented in the proximal colon and results in increased SCFA levels in this region of the gut; conversely, slow fermentable dietary fibre will reach the distal colon and modulate SCFA production at this site [7,11]. 

Many researchers have shown mixtures of multiple dietary fibre types are more beneficial than a single dietary fibre [7,12,13,14]. Rats administered diets containing guar gum or pectin produced low proportions of butyrate in comparison to rats fed mixtures of both [12,13]. Compared with control or wheat bran diets alone, diets containing a combination of wheat bran and resistant starch produced higher wet and dry output, a lower pH and ammonia, as well as lower levels of phenol [13]. An in vitro fermentation study showed that a combination of Raftilose (oligofructose) and guar gum results in higher total SCFAs than individual guar gum [14]. Following 24 h culture, the SCFA production rate from individual Raftilose or guar gum fermentation decreased, but when these fibres were combined, the production rate kept increasing, indicating that a combination of fibre sources may be more beneficial.

The inulin-type compound fructan has also been well studied for its ability to protect against colorectal cancer when included in the diet [15,16,17,18]. In combination with lycopene and probiotics, inulin induced apoptosis, and inhibited cell proliferation and aberrant crypt formation (ACF) in rat colon when the chemical 1,2-dimethylhydrazine (DMH) was used to induce cancer [19]. Inulin intake reduced CRC levels in rats fed a high lipid diet or chemical-induced CRC through decreasing enzyme activity and bile acid concentration [20,21]. The dietary fibre pectin is fermentable by human faecal bacteria and produces high proportions of acetic acid in vitro and in vivo [22]. Fermentation supernatant from incubation of human faecal slurry with apple pectin was found to be rich in butyrate and inhibited histone deacetylase in nucleus extracted from tumour cell lines [23]. 

Green cincau (*Premna oblongifolia* Merr) is a tropical plant belonging to the Verbenaceae family which is a traditional food source in Indonesia. Extracts from green leaf of the cincau plant contain about 20% pectin and have free radical scavenging activity [24]. Research on cincau extracts indicates they have the ability to induce cell-mediated immune responses in vitro [25]. As a dietary fibre, cincau extracts have laxative properties and can effectively induce the growth of lactic acid producing bacteria in the colon [26]. This study aimed to compare the efficacy of cincau extracts as a traditional source of dietary fibre with other dietary fibre combinations known to be protective against CRC. Our study found that, when fermented, cincau extracts were able to reduce Caco-2 cell viability, but the mechanism was unclear. In addition, this study found that when two different dietary fibres were combined, the benefits were not always additive. 

## 2. Materials and Methods

### 2.1. Green Cincau Extracts

Green cincau leaves (*Premna oblongifolia* Merr.) were collected from traditional farmers in Indonesia. The fresh leaves were dried in an oven at 50 °C (water content around 12%), ground into fine powder, and imported into Australia using an AQIS permit (IP07024278). To prepare extracts for this study, 5 g of dried cincau leaf powder was placed in a glass beaker, then boiled water was added until the final volume was 100 mL, and the mixture was stirred for 5 min at maximum speed. The mixture was then filtered and allowed to set at room temperature [24]. The resulting jelly like extract was then freeze dried (Dynavac) and ground with mortar and pestle before use. Table 1 shows the freeze dried cincau extract composition as determined by CSIRO analytical tests (Adelaide, South Australia), as described in Belobrajdic [27]. A modification of the AOACI Method 994.13 [28] was used to determine dietary fibre composition as soluble and insoluble non-starch polysaccharides (NSPs) [27].

### 2.2. In Vitro Fermentation of Dietary Fibre

Seven substrates as a single or a mixture of two dietary fibres (50:50) were tested following a CSIRO protocol (Adelaide, Australia): pectin, inulin, cellulose, pectin-cellulose mixture, inulin-cellulose, pectin-inulin, and green cincau extract (Table 2). The anaerobic batch fermentation was carried out as described in Charoendsiddhi et al. [29] and was adapted from Zhou et al. [30]. Briefly, 150 mg of each dietary fibre source or a 50:50 mixture was placed in a 15 mL capped tube, then 9 mL of sterile fermentation media was added. The media contained 0.25% (*w*/*v*) Tryptone, 125 ppm (*v*/*v*) micro-mineral solution (containing CaCl_2_·H_2_O 13.2%, MnCl_2_·4H_2_O 10%, CoCl_2_·6H_2_O 1%, and FeCl_3_·6H_2_O 8%), 25% (*v*/*v*) carbonate buffer solution (0.4% NH_4_HCO_3_ and 3.5% NaHCO_3_), 25% (*v*/*v*) macro-mineral solution (containing Na_2_HPO_4_ 0.57%, KH_2_PO_4_ 0.62%, and MgSO_4_·7H_2_O 0.06%), and 3.35% (*v*/*v*) reducing solution (containing cysteine hydrochloride 0.625%, Na_2_S·9H_2_O 0.625%, and NaOH 0.04 M). After addition of the dietary fibre/s to the fermentation media, the pH was adjusted to pH 7.0. For inoculums, fresh faecal slurry from three healthy volunteers was pooled and diluted in phosphate buffer to produce 10% (*w*/*v*) inoculums. Signed consent was obtained from staff volunteers for the collection of fresh faecal samples. The faecal collection process was approved by the CSIRO Human Ethics Committee. Final concentration of inoculums was 1% (*w*/*v*) after mixing of 1 mL of 10% inoculums with 9 mL of media containing dietary fibre/s. A negative control containing only fermentation media and inoculum was prepared as a faecal blank (FB). All processes were carried out in anaerobic chamber with rocking (SL Bactron IV, Cornelius, OR, USA) to maintain anaerobic conditions set at 37 °C for 24 h. To help monitor that the chamber was maintained under anaerobic conditions, 1.25 ppm (*w*/*v*) of resazurine solution (Sigma-Aldrich, St Louis, MO, USA) was added to the initial fermentation media. Supernatants were sterilized by filtration (pore size 0.22 µm) (Minisart^®^, Sartorius AG, Dandenong South, Victoria, Australia) and stored at −80 °C until use.

### 2.3. Cell Culture

Human colorectal carcinoma cells Caco-2 were obtained from the American Type Culture Collection (ATCC Number CCL-247). Experiments were conducted on Caco-2 cells passage number 76 to 85 and were performed three to four passages post thawing. Cultures were maintained in Dulbecco’s modified Eagle’s medium (DMEM) (Sigma-Aldrich) supplemented with 10% fetal bovine serum (Bovogen, Victoria, Australia), 100 U/mL penicillin-streptomycin (Sigma-Aldrich), 1% nonessential amino acids (Sigma-Aldrich), and 20 mM 4-(2-hydroxyethyl)-1-piperazineethanesulfonic acid (HEPES, Sigma-Aldrich) in a CO_2_ incubator (37 °C and 5% CO_2_). For each assay, cells were seeded so that following the 24 h attachment and 48 h experimental time, cells reached 80%–90% confluence.

### 2.4. SCFA Analysis

Fermentation supernatant (FS) samples were homogenized in three volumes of internal standard solution (heptanoic acid, 1.68 mmol/L) (Sigma-Aldrich) and centrifuged at 3000× *g* for 10 min. The supernatant was then distilled and 0.3 µL was injected into a gas chromatograph (Hewlett-Packard 5890 Series II A, Wilmington, DE, USA) equipped with a flame ionization detector and a capillary column (Zebron ZB-FFAP, 30 m × 0.53 mm i.d., 1-µm film, SGE, Phenomenex, Torrance, CA, USA). Helium was used as the carrier gas; the initial oven temperature was 120 °C and was increased at 30 °C/min to 190 °C; the injector temperature was 210 °C and the detector temperature was 210 °C. A standard SCFA mixture containing acetate, propionate, and butyrate (Sigma-Aldrich) was used for calculations, and the results are expressed as µmol/g of sample [31].

### 2.5. MTT Proliferation Assay

Caco-2 cells were seeded into a 96-well plate (Costar^®^, Corning incorporated, Corning, NY, USA) at a density of 1.5 × 10^4^ cells per well 24 h before treatment with FS (day 0) to allow adherence, then incubated for 48 h in media containing 20% FS. For standard curves, 1:2 serial dilutions were prepared to generate a standard curve of 5000–80,000 cells per well, in final volume of 100 µL [32].

After 48 h treatment, media was removed and 100 µL of medium containing 0.5 mg/mL 3-4,5-dimethylthiazol-2-yl)2,5-diphenyl-tetrazolium bromide (MTT) (Sigma-Aldrich) solution was added to each well and incubated (37 °C, 5% CO_2_) for 1 h (to allow MTT to be metabolized). The formazan (MTT metabolic product) was resuspended in 80 µL of 20% sodium dodecyl sulfate (SDS, Amresco, Solon, OH, USA) in 0.02 M HCl (Sigma-Aldrich), and the plate was incubated in the dark for 1 h at room temperature. The optical density was read at 570 nm with background absorbance at 630 nm (FLUOstar omega, BMG Labtech GmbH, Ortenberg, Germany). Optical densities were converted to a total number of live cells using a linear regression plot. Results were expressed as the number of live cells in wells containing treatment compared with the number of cells in control wells (medium alone).

### 2.6. Alkaline Phosphatase (AP) Activity Assay

For the AP assay, 3.0 × 10^5^ cells were seeded into each well of a six-well plate with supplemented media (as described above) and allowed to adhere for 24 h. The medium was removed and replaced with media containing 20% FS. After 48 h incubation, the medium was removed and cells were detached by incubation with 1× Trypsin-EDTA solution (Sigma-Aldrich) for 5 min at 37 °C. Detached cells were resuspended in 50 mM Tris-HCl buffer, pH 10.0, and homogenized by sonification. The homogenized cells were centrifuged at 100,000 rpm for 30 min to remove cell debris. 

AP activity was measured by hydrolysis of *p*-nitro phenol phosphate (5 mM) (Sigma-Aldrich) and expressed in units (the number of μmol *p*-nitrophenol liberated in 1 min measured at 400 nm per mg protein). *p*-nitrophenol (0–200 μM) was used to generate a standard curve [33].

### 2.7. Caspase 3–7 and Lactate Dehydrogenase (LDH) Assay

Caco-2 cells were seeded into 96-well white plates (Costar^®^) at a density of 1.5 × 10^4^ cells per well in supplemented media. The cells were incubated 24 h to allow the cells to adhere prior to treatment with 20% FS or control. After FS treatment, the cells were incubated for 48 h. Staurosporine 5 µM (Sigma) was used as a positive control to induce apoptosis (data not shown). The CytoTox-ONE™ Homogeneous Membrane Integrity assay kit (Promega, Madison, WI, USA) was employed to quantify the LDH enzyme activity, where 70 µL of cell culture supernatant was mixed with 70 µL CytoTox-ONE™ Reagent and shaken for 30 s, then incubated for 10 min. The stop solution (35 µL) was added to each well, and fluorescence was measured at excitation wavelength of 560 nm and an emission wavelength of 590 nm. In parallel, quantification of caspase-3/7 activities was carried out using the Caspase-GloR 3/7 assay kit (Promega). The FS treatments were also applied to separate Caco-2 cells cultured in 96-well plates for determination of cell proliferation using the MTT assay.

To confirm the role of caspase 3/7 in the cell death mechanism, FS from the inulin, cincau extract, and faecal blank fermentation were applied to the Caco-2 cells in combination with 10 µM caspase inhibitor (Ac-DEVD-CHO, Promega). Cells were seeded in 96-well plates as outlined above and the inhibitor was added 1 h preceding FS treatment.

### 2.8. Statistical Analysis

All cell culture experiments were performed on three different occasions, and the results are expressed as the mean ± standard error of mean (SEM). Statistical analysis was carried out with the statistical program SPSS version 19. One way-ANOVA with Least Significant Difference test was used. Results were considered significant if *p* < 0.05.

## 3. Results

### 3.1. SCFA Content of Dietary Fibre Fermentation Supernatant

**Figure 1:**
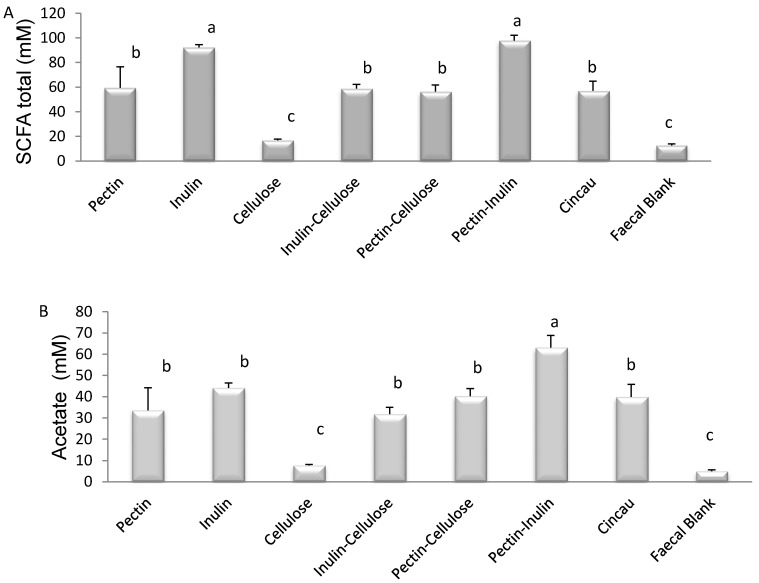

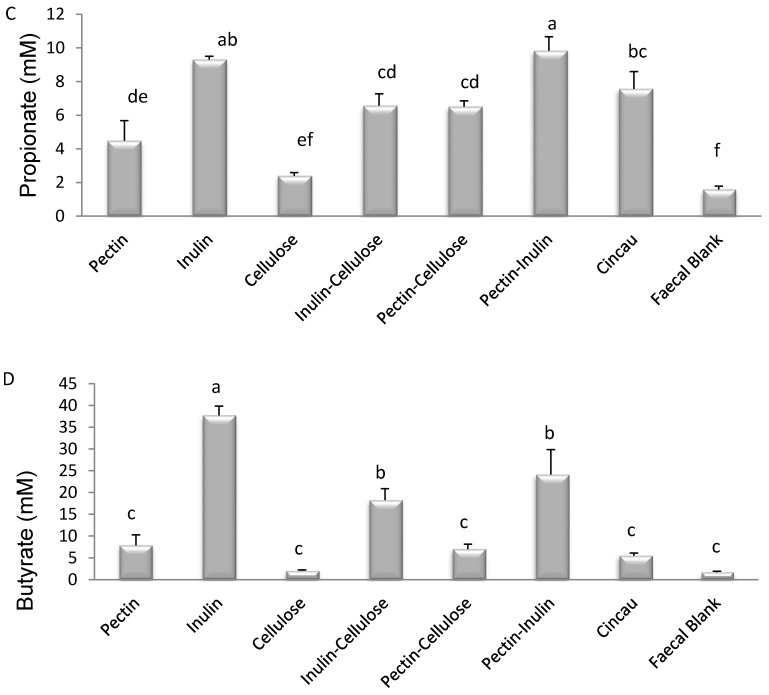
Effect of dietary fibre on the concentration of short chain fatty acid (SCFA) total (**A**), acetate (**B**), propionate (**C**), and butyrate (**D**) in fermentation supernatants. Dietary fibre/s were fermented with human faecal bacteria at 37 °C for 24 h in anaerobic conditions. The bars represent the mean, and the lines are SEM of four replicates. Data points denoted by different superscripts (letters above the bar) differ significantly when *p* < 0.05.

Fermentation with cellulose alone had no significant effect on SCFA production. In contrast, fermentation of all other dietary fibres, individually or in combination, increased the yields of total SCFA, acetate, and propionate levels in the FS in comparison to the FB (*p* < 0.05) (Figure 1A–C). Cincau as the dietary fibre source significantly increased total SCFA, acetate, and propionate, but not butyrate levels. Butyrate levels were only significantly increased in the FS after fermentation with inulin, inulin-cellulose, and inulin-pectin (Figure 1D). 

### 3.2. Effect of Dietary Fibre FS on Caco-2 Cell Viability

**Figure 2:**
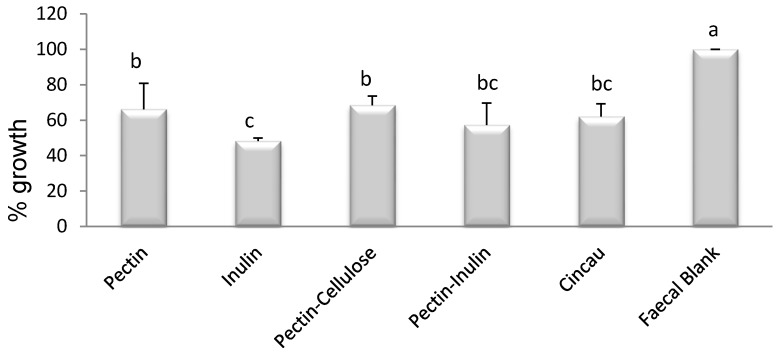
All dietary fibre sources reduced Caco-2 cell viability. Cells were seeded 1 day before the treatment with (fermentation supernatant) FS (day 0), then incubated for 48 h in media containing 20% FS. The bars represent the mean, and the lines are SEM of three independent experiments each performed in triplicate. Data points denoted by different superscripts (letters above the bar) differ significantly with *p* < 0.05.

Due to the increased production of SCFA in the FS from the different fibre fermentations, we examined the effects of the FS on Caco-2 cell viability. As cellulose alone had no effect on SCFA production and inhibited SCFA production when mixed with inulin, no further studies were performed on FS from these two dietary fibre groups. In addition, as SCFA production was no different between the faecal blank (FB) and the cellulose group, for the remaining studies the blank served as the negative control. Treatment of Caco-2 cells with the remaining five FSs affected cell viability. Caco-2 cell number was significantly reduced after incubation of cells with 20% FS after incubation with cincau and with other dietary fibre/s compared to control FB (Figure 2, *p* < 0.05). Incubation with inulin FS inhibited cell growth the most when compared to FB. Combining pectin with inulin in the FS had no significant effect on the ability of inulin or pectin to inhibit cell growth.

### 3.3. Effect of Dietary Fibre FS on Cell Differentiation

**Figure 3:**
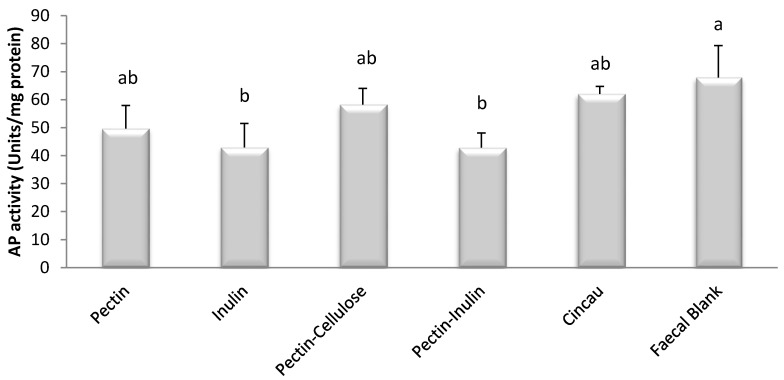
Effect of dietary fibre FS on alkaline phosphatase (AP) enzyme levels. AP enzyme activity was measured by hydrolysis of *p*-nitro phenol phosphate (5 mM) and expressed in units (the number of μmol *p*-nitrophenol liberated in 1 min measured at 400 nm per mg protein). The bars represent the mean, and the lines are SEM of three independent experiments each performed in triplicates. Data points denoted by different superscripts (letters above the bar) differ significantly with *p* < 0.05.

Cell differentiation was assessed by measuring cellular levels of the enzyme alkaline phosphatase (AP) [34]. FSs from all dietary fibre sources, including cincau, failed to increase alkaline phosphatase enzyme levels but unexpectedly some caused significant decreases in alkaline phosphatase levels (Figure 3, *p* < 0.05). Cells that were incubated in FS after fermentation with inulin and mixtures of pectin and inulin had significantly lower alkaline phosphatase activity compared to FB, whereas cells incubated with FS from pectin, mixture of pectin-cellulose, and cincau displayed similar alkaline phosphatase activities to FB (*p* < 0.05).

### 3.4. Effect of Dietary Fibre FS on Caspase 3/7 Activity

**Figure 4:**
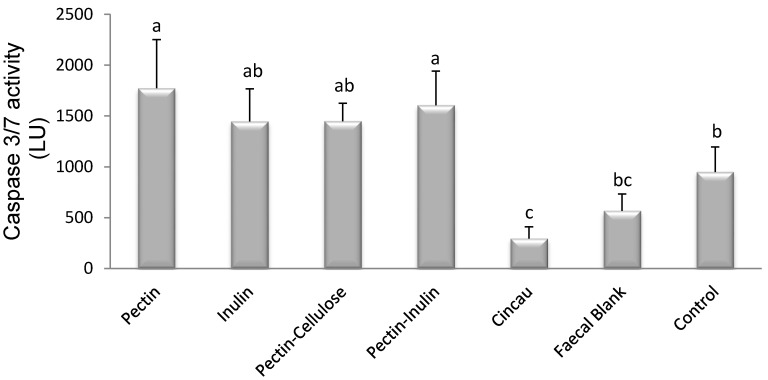
Effects of dietary fibre/s FS on caspase 3/7 activity. Cells were seeded 1 day before the treatment with FS (day 0), then incubated for 48 h in media containing 20% FS. Caspase-3 and -7 activities were measured using the Caspase-GloR 3/7 assay kit (Promega, USA). Control is cells incubated in media without FS. The bars represent the mean, and the lines are SEM of three independent experiments each performed in triplicate. Data points denoted by different superscripts (letters above the bar) differ significantly with *p* < 0.05. LU, Luminescence units.

SCFAs have been shown to reduce proliferation and induce apoptosis in colorectal cell lines [2]. Caspase 3 and 7 are key effectors of apoptosis, therefore their activity was measured in Caco-2 cells after incubation with FS. Caspase 3/7 activity was affected by the type of dietary fibre fermented by colon microbiota (Figure 4, *p* < 0.05). Pectin, individually or in combination with inulin, induced higher caspase 3/7 activity compared to no treatment (control). In contrast, cincau extracts and the faecal blank suppressed caspase 3/7 activity (*p* < 0.05).

### 3.5. Mechanism of Cell Death Induced by FSs Containing SCFAs

**Figure 5:**
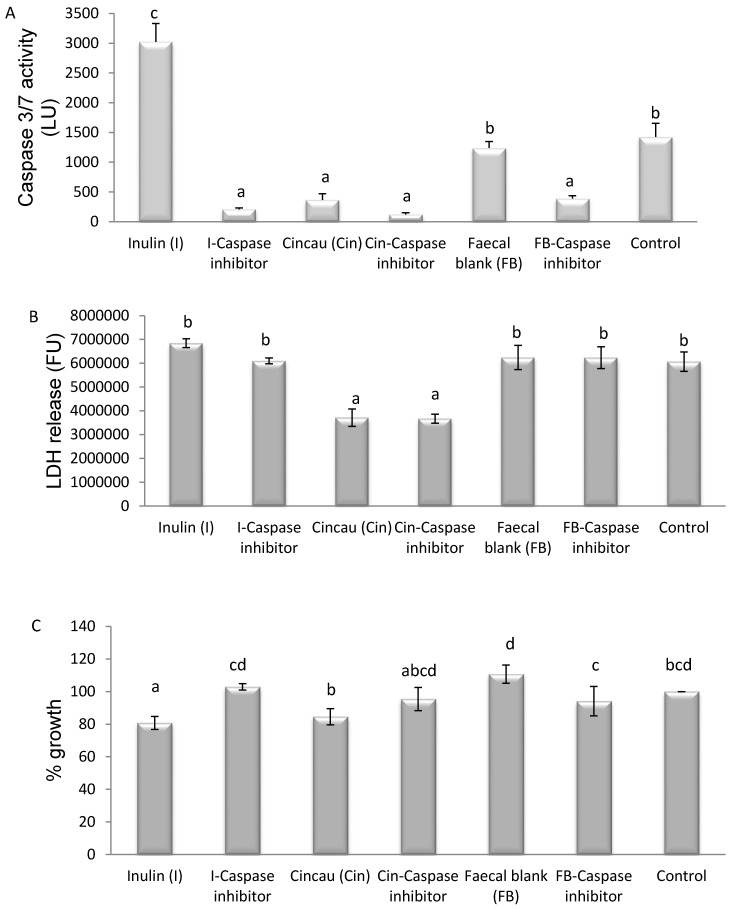
Effect of inulin and cincau on caspase 3/7 activity (**A**), LDH release (**B**), and Caco-2 cell viability (**C**) with or without caspase inhibitor. Cells were seeded 1 day before the treatment with FS (day 0), then incubated for 48 h in media containing 20% FS. Caspase inhibitor (10 µM) was added 1 h preceding FS treatment. Caspase 3–7 activity was measured using the Caspase-GloR 3/7 assay kit (Promega), the LDH activity was quantified using the CytoTox-ONE™ Homogeneous Membrane Integrity assay kit (Promega), and cell viability was measured using the MTT (dimethylthiazol-2-yl)2,5-diphenyl-tetrazolium bromide) assay and expressed as % growth against control. Control is cells incubated in media without FS. The bars represent the mean, and the lines are SEM of three independent experiments performed in triplicates. Data points denoted by different superscripts (letters above the bar) differ significantly with *p* < 0.05. LU, Luminescence units; FU, fluorescence units.

To further examine the increase in caspase 3/7 activity triggered by FSs from different dietary fibres above, a caspase inhibitor was utilised. Extracellular release of lactate dehydrogenase (LDH) was utilised as an additional measure of Caco-2 cell death. The caspase inhibitor significantly inhibited the ability of FSs from both inulin and the faecal blank to induce caspase 3/7 activity. In contrast, when cells were incubated with FS from cincau fermentation, very little caspase activity was detected and the inhibitor had no significant effect on this activity (Figure 5A; *p* < 0.05). LDH release in cells treated with FS when cincau was the fibre source was lower compared to that from inulin FS and FB (Figure 5B; *p* < 0.05). LDH release was not affected by the addition of the caspase inhibitor. Both inulin and cincau FS inhibited cell growth compared to the FB, and the caspase inhibitor was able to partially prevent this inhibition when inulin was the dietary fibre (Figure 5C; *p* < 0.05). 

## 4. Discussion

This work demonstrates for the first time that green cincau, a traditional food that is indigenous to Indonesia, can be fermented to produce SCFAs which, when tested on colon cancer cells, can inhibit cell growth in vitro. In particular, cincau fermentation resulted in increased acetate and propionate production as assessed by their concentrations, but not butyrate. Furthermore, this study demonstrates that pectin and inulin alone, or in combination, had the greatest influence on individual and total SCFA production after fermentation by gut microbiota. Inulin produced the highest concentration of butyrate among the dietary fibres tested. Butyrate levels in FS from cellulose and pectin increased significantly if these dietary fibres were mixed with inulin. Inulin is known to stimulate butyrate-producing bacteria (*Roseburia intestinalis*, *Eubacterium rectale*, *Anaerostipes caccae*), which in turn leads to higher concentrations and proportions of butyrate [35]. 

The concentration of total SCFA, acetate, and butyrate produced in culture from the cincau extract was very similar to that produced when pectin or pectin-cellulose was added as the dietary fibre. Cellulose is a non-fermentable fibre and as a result has little effect on SCFA concentrations. Given that cincau extracts are known to contain 20% pectin [24], the increases in SCFA concentrations observed may be produced by the fermentation of the pectin component of this extract. However, when analysed these hot water extracts contained 5.8% soluble NSPs and 46.3% insoluble NSPs, thus it appears that the heating, cooling, and freeze drying process may have modified chemical and physical properties of the non-starch polysaccharides including pectin to form insoluble NSPs [36]. These now insoluble NSPs may also contribute to SCFA production during the in vitro fermentation. 

SCFAs, and particularly butyrate, are well known for their ability to inhibit proliferation and induce apoptosis of colorectal cancer cells [2,3], but in this study high levels of butyrate in FS did not always affect cell viability. The butyrate content in pectin-inulin FS was nearly two-fold higher than pectin alone FS, while the propionate and acetate content from pectin-inulin FS was nearly double of pectin FS, however, the effect on Caco-2 cell growth when cultured in media containing these fermentation supernatants was no different. This indicates that butyrate or combinations of butyrate with propionate or acetate are not the main factors in fermentation supernatant that affect cell growth, and that non-SCFA compounds also contained in the FS may be involved [33]. Interestingly, cincau extract and pectin-cellulose FSs which had lower concentrations of total or individual SCFAs than pectin-inulin FS, inhibited Caco- 2 cell growth to the same extent as pectin-inulin FS. 

Cell differentiation is one of the mechanisms by which SCFAs act in order to slow cancer cell growth [37]. This process requires cells to enter G1/G0 phase arrest, and cell proliferation is then inhibited [38]. Our results indicate that FS from pectin, inulin, pectin-cellulose mixture, pectin-inulin mixture, or cincau extract do not induce alkaline phosphatase, a marker of cell differentiation. Surprisingly, FS from blank (FB) induced higher alkaline phosphatase levels than pectin, inulin, or pectin-inulin, even though all of these dietary fibre FSs had high SCFA concentrations. There are some possibilities to explain these observations. First, the effect of butyrate on cell differentiation is dose dependent. It was previously observed that butyrate induced cell differentiation of Caco-2 cells at a concentration of 0.1 mM, but when the butyrate level was increased to 5 mM, activity of this enzyme decreased [39]. In the present study, the inulin and pectin-inulin FSs contained 37.7 and 24.2 mM of butyrate, respectively. When 20% of these FSs were added to the media, the final concentration of butyrate in the media would be 7.5 and 4.8 mM, respectively, whereas the final concentration of butyrate in media containing pectin, pectin-cellulose, and cincau was only 1.6, 1.4, and 1.1 mM, respectively. It is suggested that high levels of butyrate in inulin and pectin-inulin FSs may have led to the downregulation of AP activity. However, this explanation is not likely, as the blank (FB) which contains very little SCFA, elicited higher AP levels. Therefore, a second possibility needs to be considered to rationally explain the effects of FB. Previous researchers have also found that FB had an effect that was unexplainable by SCFA content in FS [16,33,40]. For example, Sauer et al. found that metabolic activity of HT-29 cells was increased by 15% by FB supplementation, with levels increasing similarly to those from inulin FS [16]. Moreover, these authors also found that FB enhanced gene expression of GSTA4, but inulin FS or SCFA mixture had no effects on this gene. GSTA4 is a gene encoding a glutathione S-transferase belonging to the alpha class 4 that has high catalytic efficiency with 4-hydroxyalkenals and other cytotoxic and mutagenic products of radical reactions and lipid peroxidation [41]. Taking this into account, data from this study indicate that the effect of FSs from dietary fibre on cell differentiation may depend on several factors including SCFA pattern and unidentified products formed during the fermentation process or that originally exist in the fresh faecal sample as a source of inoculums. 

The ability of pectin, inulin, pectin-inulin, and pectin-cellulose FSs to induce apoptosis was confirmed by their ability to increase caspase 3/7 activity compared to control (Figure 4; *p* < 0.05). In contrast, cincau and FB, when compared to control, decreased levels of caspase 3/7. Previous research has shown that inulin induced apoptosis in HT-29 cells [42] or in a colon cancer rat model [43]. Our results support that inulin or pectin-inulin FSs are able to induce apoptosis through caspases, as caspase 3/7 activity increased in Caco-2 cells incubated with these FSs (Figure 4, *p* < 0.05). 

Pectin or pectin-cellulose mixtures also increased caspase 3/7 activity (Figure 4, *p* < 0.05), and this may support a role for pectin via its increase in SCFAs as a dietary fibre that can affect the apoptosis process. Butyrate or other SCFAs produced by the fermentation of pectin may be able to inhibit histone deacetylase activity in order to induce gene transcription of caspase 3 and induce apoptosis [6,44,45,46].

Butyrate is the most potent SCFA for modulating colorectal cancer growth, including the induction of apoptosis [6,47]. However, our data indicate that modulation of apoptosis is not always dependant on butyrate content. The effect of FSs on caspase 3/7 activity was also unexplainable by total SCFA content of FS. Therefore, some factors other than SCFAs might be involved in modulating caspase 3/7 activity [23,42].

FSs from inulin, cincau, and FB were chosen to further elucidate the role of caspase 3/7 on cell death using the caspase inhibitor (Ac-DEVD-CHO) before the application of FS. LDH is an accurate method to assay cell death with membrane damage such as necrosis, while the MTT assay can measure differences in cell viability, but it cannot tell whether cells are being killed via apoptosis or necrosis [45]. The FS from inulin induced cell death through a caspase 3/7-dependent pathway, as the release of caspase 3/7 could be inhibited by the addition of the caspase inhibitor, and this led to an observed increase in Caco-2 cell viability. Cincau extract FS suppressed Caco-2 cell growth compared to FB (Figure 5C, *p* < 0.05), but the mechanism appeared to be different to that observed with inulin. Compared to FB, cincau did not induce caspase 3/7 activity (Figure 5A, *p* < 0.05), and indeed less LDH was released from cells treated with either cincau or cincau and caspase inhibitor (Figure 5B, *p* < 0.05), suggesting that cincau could protect cells from necrotic cell death. However, these cells were less viable than the control cells. Previously, Huang et al. [48] found that *Solanum nigrum* Linn leaf extract, rich in polyphenols and anthocyanidin, caused cell death due to the induction of autophagy and apoptosis. Acetone and ethyl acetate extracts from *Eupatorium odoratum* induced autophagic cell death in MCF-7 and Vero cell lines [49]. Cincau was extracted from green cincau leaves (*Premna oblongifolia* Merr.). The extract contains alkaloids, saponins, phenol hydroquinones, molisch, benedict, and tannins [50]. Supernatants collected after the non-digestible fraction of cooked common bean (*Phaseolus vulgaris* L.), when fermented with gut microbes, were able to induce apoptosis of HT-29 colon cells, and this was thought to be due to the participation of other phenolic fatty acid derivatives and biopeptides and not the SCFA contained in the supernates [51] Therefore, it may be possible that the phytochemical compounds from cincau or cincau fermentation induce autophagic cell death which cannot be measured by either the caspase or LDH assay, but would be worth investigating in future studies.

Our research implies that the beneficial effects of mixed dietary fibre as experienced in most human diets will depend on how each dietary fibre consumed interacts with the colon microbiota, and suggests the important role of unidentified compounds produced during fermentation by gut microbes in modulation of the effect of dietary fibre on CRC carcinogenesis. Furthermore, for the first time we show that Green cincau, a traditional Indonesian food, is not only able to inhibit colon cancer cell growth, but by an apoptosis-independent pathway. Further work should be conducted to assess the ability of this novel traditional dietary fibre as a chemopreventative.

## Figures and Tables

**Table 1:** Composition of dried green cincau extract (g/100 g dry weight).

Dietary Component	Concentration
Moisture	4.4
Fat	4.4
Protein	13.3
Ash	12.2
Starch	1.8
Resistant starch	0.5
Soluble NSP	5.8
Insoluble NSP	46.3
Total non-starch polysaccharides (NSPs)	52.1

**Table 2:** Type of dietary fibre and combinations used for batch in vitro fermentation.

Dietary Fibre	Ratio (%)
Pectin	100
Inulin	100
Cellulose	100
Pectin + cellulose	50:50
Pectin + inulin	50:50
Inulin + cellulose	50:50
Cincau extract	100
Faecal blank (FB)	-
